# Supplementary material for: A Global Screen for Assembly State Changes of the Mitotic Proteome by SEC-SWATH-MS
Source: Cell Syst. 2020 Feb 26;10(2):133–155.e6. doi: 10.1016/j.cels.2020.01.001 (PMC7042714; doi:10.1016/j.cels.2020.01.001)

P18583 | SON_HUMAN | SON C21orf50 DBP5 KIAA1019 NREBP HSPC310 HSPC312
Monomer MW [kDa]: 263.83 Monomer expected elution fraction: 32

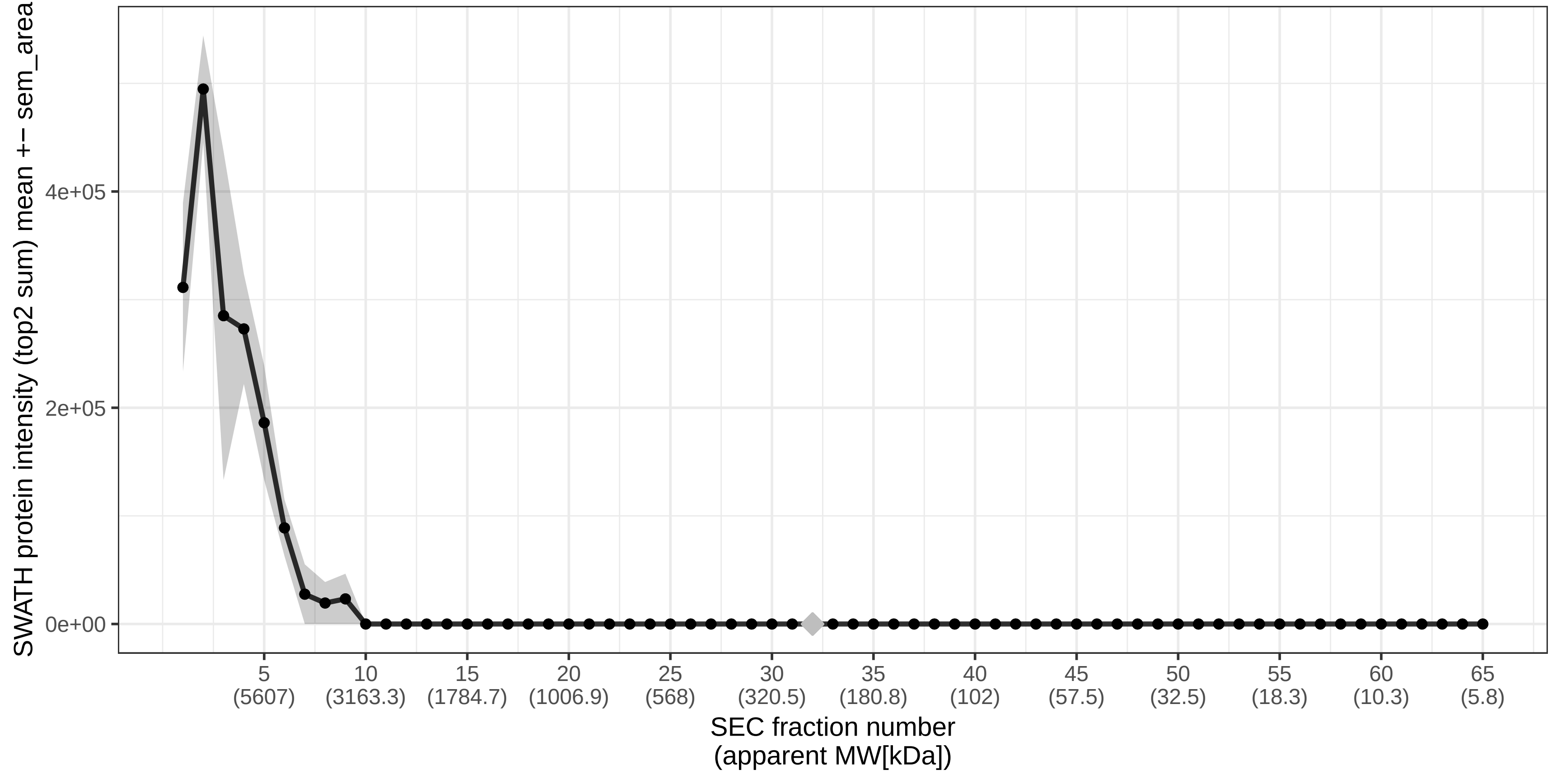

Supplement: Data S1. SEC-SWATH-MS Protein Chromatograms, Related to Figure 1 [file mmc6.zip › SECchrom_P18583_SON_HUMAN_SON_C21orf50_DBP5_KIAA1019_NREBP_.pdf]
